# A high fat, sugar, and salt Western diet induces motor‐muscular and sensory dysfunctions and neurodegeneration in mice during aging: Ameliorative action of metformin

**DOI:** 10.1111/cns.13726

**Published:** 2021-09-12

**Authors:** Song Hong, Aarti Nagayach, Yan Lu, Hongying Peng, Quoc‐Viet A. Duong, Nicholas B. Pham, Christopher A. Vuong, Nicolas G. Bazan

**Affiliations:** ^1^ Neuroscience Center of Excellence Louisiana State University Health Sciences Center New Orleans Louisiana USA; ^2^ Department of Ophthalmology Louisiana State University Health Sciences Center New Orleans Louisiana USA; ^3^ Biostatistics Department of Environmental Health University of Cincinnati College of Medicine Cincinnati Ohio USA

**Keywords:** amyloid beta and phosphorylated tau in Alzheimer's disease, metformin, microglia in neuroinflammation, neurodegeneration in motor and sensory cortexes, Western diet combing high saturated fat, sugar and salt

## Abstract

**Aims:**

To explore the novel linkage between a Western diet combining high saturated fat, sugar, and salt (HFSS) and neurological dysfunctions during aging as well as Metformin intervention, we assessed cerebral cortex abnormalities associated with sensory and motor dysfunctions and cellular and molecular insights in brains using HFSS‐fed mice during aging. We also explored the effect of Metformin treatment on these mice.

**Methods:**

C57BL/6 mice were fed with HFSS and treated with metformin from 20 to 22 months of age, resembling human aging from 56 to 68 years of age (an entry phase of the aged portion of lifespan).

**Results:**

The motor and sensory cortexes in mice during aging after HFSS diet showed: (A) decreased motor‐muscular and sensory functions; (B) reduced inflammation‐resolving Arg‐1^+^ microglia; (C) increased inflammatory iNOs^+^ microglia and TNFα levels; (D) enhanced abundance of amyloid‐β peptide and of phosphorylated Tau. Metformin attenuated these changes.

**Conclusion:**

A HFSS‐combined diet caused motor‐muscular and sensory dysfunctions, neuroinflammation, and neurodegeneration, whereas metformin counteracted these effects. Our findings show neuroinflammatory consequences of a HFSS diet in aging. Metformin curbs the HFSS‐related neuroinflammation eliciting neuroprotection.

## Graphical abstract

This report reveals for the first time that Western diet combining high saturated fat, sugar, and salt (HFSS) reduces the motor‐muscular grip strength and sensory responsibility of mice in aging, which corresponds to increases of neurodegeneration, neuroinflammation (the switch from M2 to M1 phenotype of microglia and increased TNFα levels), pTau burden, and Aβ deposition in motor and sensory cortexes. Metformin counteracts the key portion of HFSS effects. These data address the knowledge gap of neurodegenerative consequence of HFSS diet and involved cellular and molecular insights.

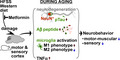

## 1 INTRODUCTION

The “Western” diet that combines high saturated fat, sugar, and salt (HFSS) is an important risk factor for many chronic disorders, including stroke, diabetes, hypertension, obesity, coronary artery disease, cognitive impairments, and Alzheimer's disease (AD).^1^, ^2^, ^3^, ^4^, ^5^, ^6^ Consumption of a Western diet with a single or double high feature of fat, sugar, and salt exacerbates metabolic disturbances^7^, ^8^, ^9^, ^10^ and oxidative stress in the entire body,^7^, ^9^, ^10^, ^11^, ^12^ and is associated with microglial activation in the cortex, as well as motor function disturbances.^13^ Epidemiology has shown that diet has profound effects on neurodegeneration‐involved aging.^14^ Complications induced by a Western diet increase the risk of early development of aging‐associated brain disorders.^15^, ^16^ Long‐term consumption of a high salt diet induces somatosensory and cognitive dysfunctions,^4^, ^17^, ^18^ while exacerbating age‐associated cognitive and behavioral dysfunctions by affecting the brain redox balance.^17^

Metformin (Met) (1,1‐dimethylbuguanide hydrochloride) has the potential for treating brain disorders and is effective in managing metabolic disorders, including diabetes and hypertension,^19^, ^20^, ^21^ through its insulin‐sensitizing effect, which lowers glucose and free fatty acid levels. Met is also a potentially effective treatment for Parkinson's disease,^22^ ischemic brain disease,^23^ diabetes‐allied dementia and cognitive decline,^24^, ^25^, ^26^ Huntington's disease,^27^ and AD.^28^ Met also attenuates aging‐associated neurodegeneration.^29^, ^30^, ^31^ Therefore, Met could be used to curb neurodegenerative disorders associated with the combination of diet and aging.

Many reports have described the unhealthy effects of a single or double high feature of fat, sugar, and salt in Western diets. However, Western diets are usually high in all three of these components. To address this vital gap in knowledge, we assessed here the effects of a HFSS diet on motor‐muscular and sensory behaviors, as well as on neuronal loss by Western blotting and by immunohistological measurement of the levels of the M1‐like inflammatory and M2‐like inflammation‐resolving phenotypes of microglia cells in the cerebral cortex.^32^, ^33^, ^34^, ^35^, ^36^ We also explored the effects of HFSS on the accumulation of phosphorylated tau (pTau) and beta‐amyloid (Aβ), which are the biomarkers and postulated causes of AD pathogenesis.^4^, ^16^, ^18^, ^33^, ^37^, ^38^, ^39^, ^40^, ^41^ In addition, we determined whether metformin treatment would ameliorate any deleterious effects of HFSS diet.

Peripheral sensory behavior is controlled by the sensory cortex, while the motor cortex is critical for controlling muscle reflexes and strength.^42^, ^43^ We used a novel approach that incorporated consumption of HFSS by C57BL/6 mice from 20 to 22 months of age. This age approximates human aging from 56 to 68 years of age,^44^, ^45^, ^46^, ^47^ an apparently critical entry phase of the aged portion of the human lifespan that is likely to be important in the neurodegeneration associated with the combination of HFSS and aging. The results of this study provide mechanistic insights into microglial phenotypes and suggest the intervention using metformin to curb the onset of neurodegenerative diseases.

## 2 MATERIALS AND METHODS

### 2.1 Animals

The Louisiana State University Health Science Center (LSUHSC) IACUC committee approved all the animal procedures. C57BL/6 (B6) mice were maintained at LSUHSC at a controlled temperature of 25 ± 2°C and 50%–65% humidity with a fixed 12:12 h light‐dark cycle. The animal data report has followed the ARRIVE guidelines.^48^

### 2.2 HFSS Western diet and metformin treatment

Twenty‐month‐old male B6 mice were randomly separated into three groups (*n* = 4 in each group): (A) normal diet (ND); (B) HFSS diet; and (C) HFSS with metformin injection (Sigma, 100 mg/kg body weight) (HFSS + Met). Mice in the HFSS and HFSS + Met groups were fed a diet (ad libitum) with high fat [41% of the total kcal (44% saturated and 56% unsaturated)], high refined sugar [39% kcal (27% kcal sucrose +12% kcal maltodextrin‐10) sugar], and 0.25% salt (NaCl by weight) (D12079B, Research Diets, Inc.), as well as a high salt water drink (1% NaCl for 7 weeks, followed by 4% salt for 4 days and then 3% salt for a week). The ND consisted of 10% kcal fat, 73% kcal carbohydrate (corn starch, 82.2% plus maltodextrin‐10%, 17.8%, by weight), 17% kcal protein, and 0.25% salt, and tap‐water drink contained <0.02% salt. Our design of the HFSS diet was based on previous studies on Western diets, although all reported studies focused on the single or double high feature of fat, simple sugar, and salt.^4^, ^18^, ^37^, ^38^, ^39^, ^49^, ^50^ All the mice were separated into two groups and fed either the ND or HFSS diet for two months. During the second month of the diet, one set of mice from the HFSS group was treated with metformin (Sigma, 100 mg/kg body weight, *i*.*p*., every two days for a month) and designated as the HFSS + Met group.

### 2.3 Loaded grid test

This test is based on the tendency of a mouse, when suspended by the tail, to grasp a grid with its forelimbs.^51^ Here, weights of specific mass are hung from the grid, and the total time the mouse held a given weight was used as an assessment of motor‐muscular strength. Mice were habituated for 60 min before the tests. Prior to the test, the body weight of the mouse was recorded to allow normalization of the body weight. The mouse was held by its tail and allowed to grasp the weighted grid. Trials were provided with different weight measures. A resting period of 5 min was provided for each mouse between each series of pulls to allow recovery and to avoid habit formation.

### 2.4 Hot water tail flick test

This test evaluates the animal's sensitivity to a hot temperature.^52^ The mice were habituated to the experimental setting for 60 min. A 500 ml glass beaker filled with 450 ml distilled water was warmed on a hot plate to the specified temperatures (*ie*, 42 and 55°C). The water temperature was monitored with a glass thermometer and maintained throughout the experiment. Approximately 5 min before the test, the mouse was removed from the cage and allowed to crawl into a precut 50 ml tube. The tail was marked with waterproof ink 3 cm from the distal tip. The tube holding the mouse was held in a horizontal position to allow the distal 3 cm of the tail to be submerged in the preheated water bath. The tail submersion time was limited to 30 s. The flicking movement of the tail out of the water was recorded as the tail flick time and recorded accordingly. Each mouse underwent four trials, with a 10 min interval between each trial.

### 2.5 Tissue collection and processing for immunohistochemistry

After behavioral assessments, mice were anesthetized with ketamine and xylazine and then sacrificed through decapitation. One cerebral hemisphere of each mouse was preserved in 4% paraformaldehyde and then cryoprotected in phosphate‐buffered sucrose gradients (10%, 20%, and 30%) at 4°C until the tissue settled at the bottom. After processing, coronal sections of the motor and sensory cortex were sequentially cut at a thickness of 15 µm using a cryostat (Shandon Cryotome SME). The sections were collected serially on Superfrost slides (Fisher Scientific) and stored at −20°C until used for immunohistological studies.^53^

### 2.6 Immunohistochemistry

The co‐immunolabeling was done as previously described.^53^, ^54^, ^55^ Briefly, the tissues were permeabilized with 0.5% Triton X‐100, and non‐specific proteins were blocked with 3% normal serum (dissolved in washing buffer). The sections were then washed three times with washing buffer for 5 min each and incubated overnight at 4°C with the following primary antibodies: Iba‐1 (1:500; Rabbit polyclonal; FujiFilm), Arg‐1 (1:500; mouse monoclonal; Santa Cruz), iNOs (1:500; Rat monoclonal; Invitrogen), pTau (1:500; Rabbit polyclonal; pSer199/202, Cat.# 44‐768G, ThermoFisher Scientific), NeuN (1:500; mouse monoclonal, Cat. #104224, Abcam), Amyloid beta (Aβ; 1:4000; MOAB‐2, mouse monoclonal, Millipore), and collagen‐IV (Col‐IV, 1:500; rabbit polyclonal; BioRad). For double labeling, cocktails were prepared that kept the different host species of the primary antibodies under consideration. The sections were then given three washes and incubated for 90 min at room temperature with the appropriate secondary antibodies, including Alexa 488 and Alexa 568 (1:500 for each; Invitrogen). The sections were then given four 10 min rinses with buffer and mounted with aqueous mounting media (Aqua Mount; Lerner Laboratories). Comparable immunostaining was ensured by processing all the sections together at the same time under the same conditions. The primary antibodies were omitted in the negative controls.

### 2.7 Cell quantification and analysis

Cell quantification was done on sections stained with the standard immunofluorescence method.^53^, ^54^, ^55^, ^56^, ^57^, ^58^, ^59^ The cell populations per microscope field were estimated in the motor and sensory cortexes. Equal areas were assessed in every section by applying an 1162.5 µm frame encompassing the anatomical areas of interest to ensure that counts were representative of the analyzed areas. Microimages of the motor and sensory cortex regions located between bregma 0.74 mm and 0.02 mm, respectively,^60^ were photographed with a Zeiss LSM710 confocal microscope. Only tissue sections corresponding to those coordinates were included in the quantification to ensure that the regions of interest were equivalent among animals and experiments. Co‐localization and cell counts were analyzed on 20× and 40x magnification images. Two proteins (one stained green and the other stained red) were considered as co‐localized at the section sites that displayed yellow from the superimposition of the red and green light. Nuclei were stained blue with DAPI. Cell population percentages and levels of specific proteins (labeling intensity integrated) were analyzed on the digital images using the NIH Image J software and specific plugins (http://rsb.info.nih.gov/ij/download.html).^54^, ^55^ Iba‐1^+^ microglia that were Arg‐1^+^ or iNOs^+^ were counted under the confocal microscope. The measurement settings for every sample were kept the same throughout the quantification.

### 2.8 Western blotting analysis

The other hemisphere of the brain was snap‐frozen, homogenized, and lysed in buffer containing protease and phosphatase inhibitors (ThermoFisher) and analyzed by Western blotting, as described.^61^, ^62^ In brief, the lysates were centrifuged at 14,000 rpm for 25 min at 4°C. Supernatant protein concentrations were then determined using the BCA method (Cat. #23227, Thermo Scientific), and the samples were then boiled for 5 min in Laemmli's sample buffer (Cat. # 1610737, Bio‐Rad). Equal amounts of protein were separated on SDS‐polyacrylamide gels (Cat. Cat No: #4561105, Bio‐Rad) and then transferred to polyvinylidene fluoride membranes (Cat. No: IPVH00010, Immobilon‐P, Thermo). The membranes were then blocked with TBST solution (50 mM Tris‐HCl, pH 7.5, 150 mM NaCl, and 0.1% Tween 20) containing 5% non‐fat dry milk at 4°C for 1 h. The membranes were then incubated overnight at 4°C with anti‐NeuN (1:1000; mouse monoclonal, Cat. #104224, Abcam) and anti‐GAPDH (1:1000; Cell Signaling, 2118S) in 3% BSA. After washing, the membranes were incubated with secondary antibody diluted in 3% BSA. Blots were imaged using a ChemiDoc detection system (BioRad), and signals were quantified using Image Lab software (BioRad).

### 2.9 Statistical analysis

Data were analyzed by Kruskal‐Wallis (nonparametric) test followed by Dunn test for the assessment of significant differences between groups in case data that do not have normal distribution.^63^, ^64^ The *p*‐values were adjusted by Holm method. Data were reported as dots and lines (medians) in figures or as medians in text. The correlations between specific microglia abundancies and Aβ or pTau levels in the motor or sensory cortex were analyzed via Spearman's nonparametric method.^63^, ^64^ RStudio (Version 1.4) was used. A *p*‐value ≤0.05 was considered statistically significant.

## 3 RESULTS

### 3.1 HFSS decreases motor‐muscle grip strength

**FIGURE 1:**
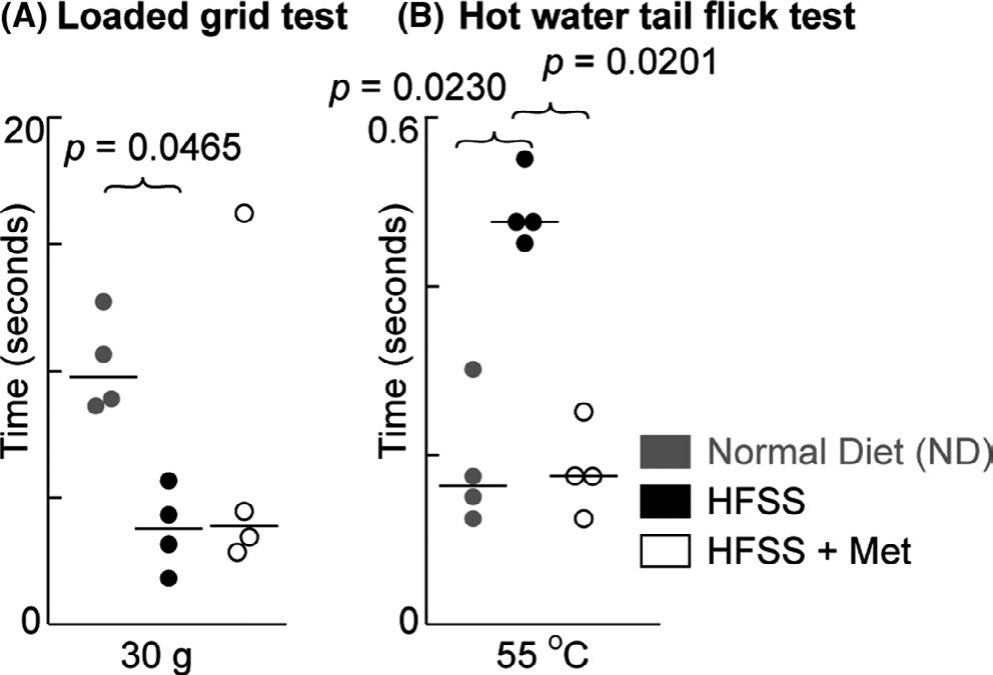
Chronic HFSS consumption and Met on the motor‐muscular grip strength and sensory behavior of mice during aging. A, HFSS‐fed mice had a significant reduction in their motor‐muscular grip strength (lifting a 30 gram weight) compared with ND‐fed age‐matched mice. B, tail flick test. HFSS consumption reduced the mouse heat sensitivity at 55°C compared with the ND control, as mice took more time to respond to the hot temperature. Following Met treatment, the HFSS‐fed mice showed improvement in their sensitivity. Data are displayed as dots and lines (medians). *p* ≤ 0.05 is significant (*n* = 4 mice)

This strength measured by loaded grid test was used to assess the motor‐muscle functions^51^ that was hypothesized to be affected by HFSS. The motor‐muscle grip strength was lower in HFSS‐fed than in age‐matched ND‐fed mice (Figure 1A) when lifting a 30 g (*p* = 0.0465) weight. No significant difference was observed for lifting a 10, 20, or 40 g weight (data not shown). Met treatment did not show effect on the muscle strength in HFSS‐fed mice (Figure 1A).

### 3.2 HFSS reduces heat sensitivity

We tested the prediction that HFSS attenuates the sensory function by measuring the heat sensitivity of mice. The tail flick test with 55°C water revealed a significantly reduced heat sensitivity of the HFSS‐fed mice compared with age‐matched ND‐fed mice (*p* = 0.0230), as the HFSS‐fed mice took a longer time to respond to the heated water (Figure 1B). Met treatment improved the heat sensitivity behavior in HFSS‐fed mice compared with the untreated HFSS‐fed mice (*p* = 0.0201), indicating a protective effect of the drug. No differences were observed with the 42°C water test (data not shown).

### 3.3 Metformin enhances cerebral neuronal density in the aging mice fed with HFSS diet

**FIGURE 2:**
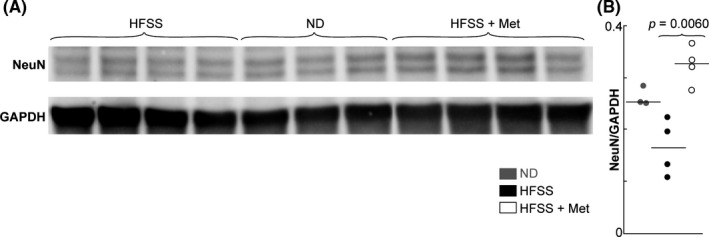
Met treatment enhanced cerebral neuronal density in aging mice fed with HFSS. A, Western blotting images of NeuN in cerebral hemispheres of HFSS‐fed mice. B. NeuN/GAPDH optical density ratios of NeuN bands over GAPDH bands. The mice during aging were treated with HFSS and/or Met. Normal diet (ND) is a control. Data are presented as dots and lines (medians). *p* ≤ 0.05 is significant (*n* = 3–4 mice)

The neuroprotective action of Met was expected to counteract the neurodegenerative potential of HFSS, thus was explored. The relative density of neurons assessed by NeuN by Western blotting in the brains of aging male B6 mice displayed a tendency to decrease in the HFSS group (*p* = 0.1302) (Figure 2). However, Met treatment enhanced NeuN abundance in the brains of HFSS‐fed mice (*p* = 0.0060) (Figure 2B).

### 3.4 Microglial responses to HFSS in the motor and sensory cortexes of aging mice

**FIGURE 3:**
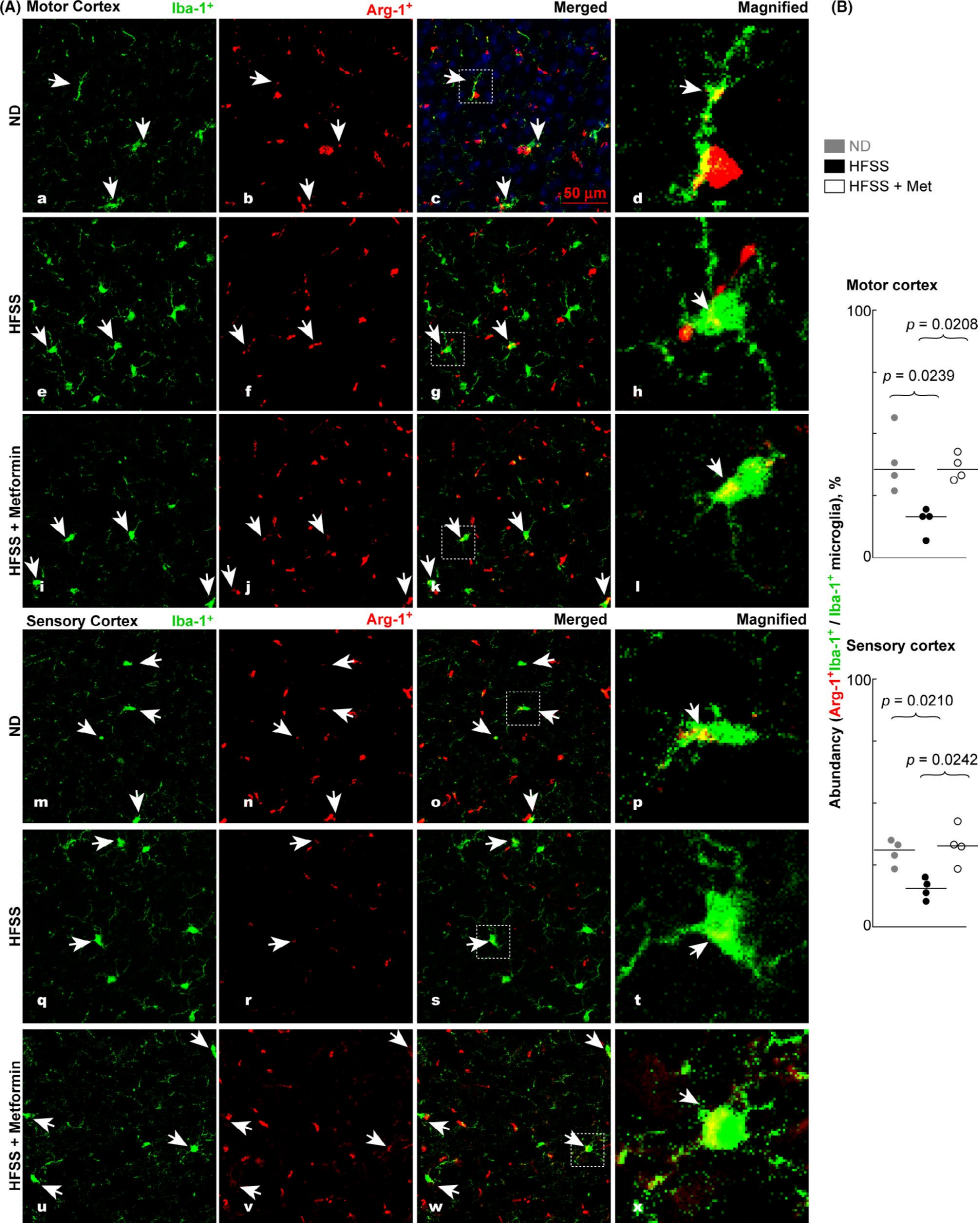
HFSS diet decreased Arg‐1‐colabeled Iba‐1^+^ microglia in the motor and sensory cortexes, whereas Met reversed this trend. A, Representative microimages of Arg‐1‐colabeled Iba‐1^+^ microglia in the motor and sensory cortexes in brain cryosections of aging B6 mice fed with ND or HFSS and treated with or without Met. B, Abundancy, M2‐like Arg‐1^+^Iba‐1^+^ microglia /total Iba‐1^+^ microglia (Arg‐1^+^Iba‐1^+^/Iba‐1^+^ microglia), % in the microscope field of the motor and sensory cortexes. Arrows annotate microglial cells with reprehensive morphology. The magnified images at the most right column of “A” show the morphology and expression of Arg‐1 (yellow) of Iba‐1^+^ microglia. Data are presented as dots and lines (medians). *p* ≤ 0.05 is significant (*n* = 4 mice). Scale bar =50 µm

Arginase‐1 (Arg‐1) is a commonly used marker for localizing alternatively activated (*ie*, M2 phenotypic) microglia and macrophages that are inflammation‐resolving and reparative.^32^, ^33^ In the ND‐fed mice, Arg‐1 was normally expressed in the motor and sensory cortexes of aging mice, and it co‐localized with some Iba‐1 protein of the Iba‐1^+^ microglia processes and cell bodies. The microglia that expressed Arg‐1 are M2‐type like Iba‐1^+^Arg‐1^+^ microglia (Figure 3A a‐d, m‐p). HFSS feeding caused a decline in Arg‐1 expression and reduced the co‐labeling of Arg‐1 in microglia cells, indicating a detrimental effect of HFSS on the cortexes (Figure 3A e‐h, q‐t) of aged mice. Met treatment increased Arg‐1 expression and co‐labeling with Iba‐1 in the cortexes of HFSS‐fed (Figure 3 i‐l, u‐x) mice, indicating a neuroprotective effect of Met against detrimental HFSS consumption‐related and aging‐associated changes.

HFSS group had lower abundancy of Arg‐1^+^Iba‐1^+^ (M2‐like) microglia over total Iba‐1^+^ microglia (*ie*, Arg‐1^+^Iba‐1^+^/Iba‐1^+^ microglia, %) than ND group in motor (16.7% *vs*. *35*.*9%*, *p* = 0.0239, Figure 3B upper right) and sensory (15.5% *vs*. *31*.*0%*, *p* = 0.0210, Figure 3B lower right) cortexes. This HFSS effect was attenuated by Met treatment as HFSS + Met group had 35.7% of Arg‐1^+^Iba‐1^+^/Iba‐1^+^ microglia in motor cortexes (*p* = 0.0208 *vs*. HFSS) and 32.9% of Arg‐1^+^Iba‐1^+^/Iba‐1^+^ microglia in sensory cortexes (*p* = 0.0242 *vs*. HFSS). These data indicated that the relative abundancy of inflammation‐resolving, reparative Arg‐1^+^ microglia was reduced in the motor and sensory cortexes by HFSS, but the Met treatment curbed this decline.

**FIGURE 4:**
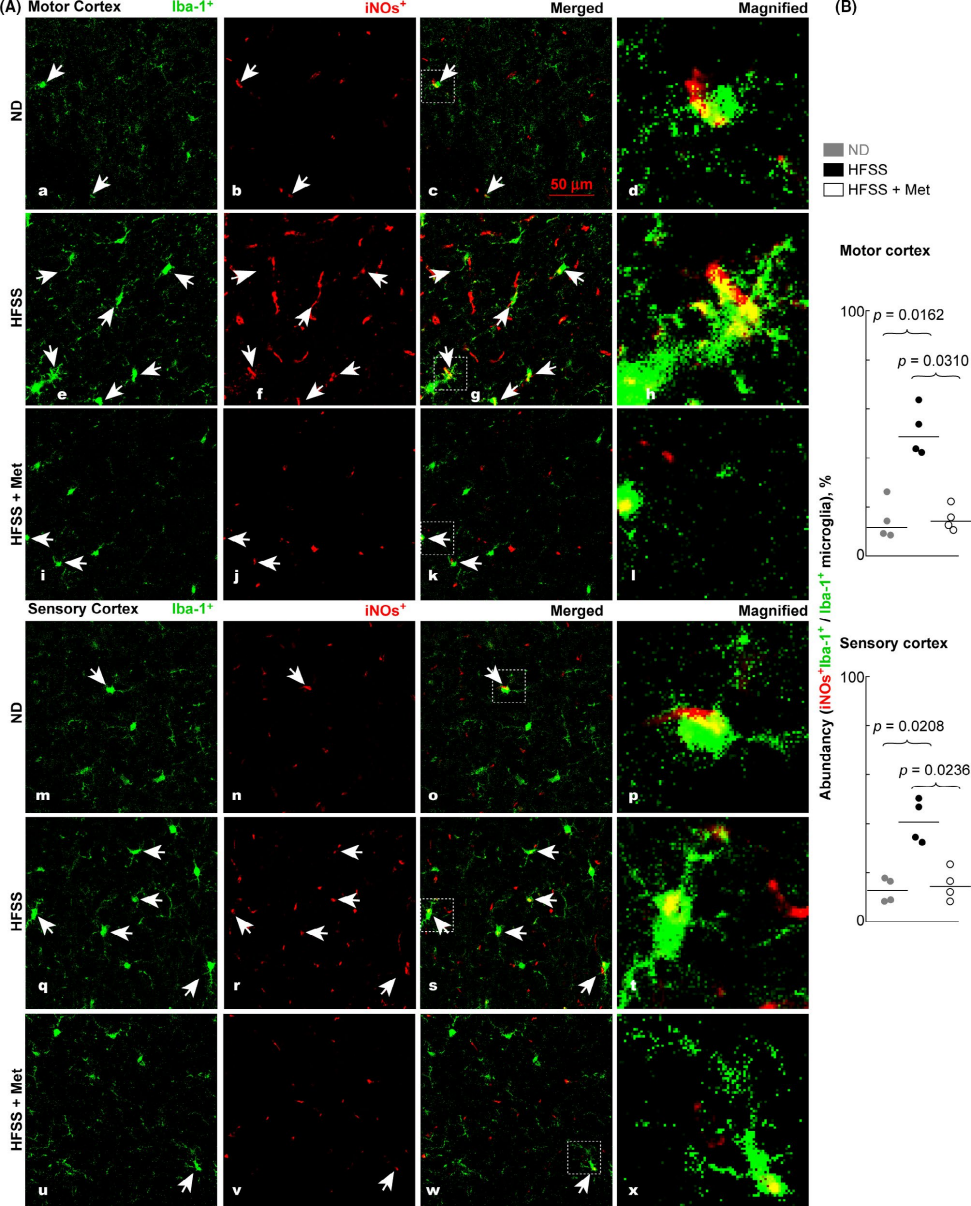
HFSS diet increased the level of iNOs‐colabeled Iba‐1^+^ microglia in the motor and sensory cortexes, whereas Met reversed this effect. A, Representative microimages of iNOs‐colabeled Iba‐1^+^ microglia in motor and sensory cortexes in brain cryosections of mice during aging after treatment with ND or HFSS and/or Met. B, Abundancy, M1‐like iNOs^+^Iba‐1^+^ microglia /total Iba‐1^+^ microglia (iNOs^+^Iba‐1^+^/Iba‐1^+^ microglia), % in the microscope field of the motor and sensory cortexes. Arrows indicate microglial cells with representative morphology. The magnified images at the farthest right column of “A” show the morphology and expression of iNOs (yellow) of microglia. Data are presented as dots and lines (medians). *p* ≤ 0.05 is significant (*n* = 4 mice). Scale bar =50 µm

Another protein biomarker that is used to characterize microglia is inducible nitric oxide synthase (iNOs), which is viewed as a marker for the M1‐like (classical activation/mediator of inflammatory response) phenotype.^33^ The HFSS‐fed mice showed an increase in iNOs‐colabeled microglial cells (Figure 4A e‐h, q‐t) compared with the ND‐fed (Figure 4Aa‐d, m‐p) and the Met‐treated (Figure 4A i‐l, u‐x) groups. The expression of iNOs‐colabeled Iba‐1^+^ microglia was reduced following Met treatment in HFSS‐fed mice (Figure 4A i‐l, u‐x). HFSS augmented the abundancy % of iNOs^+^Iba‐1^+^ microglia over total Iba‐1^+^ microglia in the motor (from 11.7% for ND to 48.5% for HFSS, *p* = 0.0162, Figure 4B upper) and sensory (from 12.9% for ND to 40.8% for HFSS, *p* = 0.0208, Figure 4B lower) cortexes. This HFSS effect was damped by Met in motor (from 48.5% for HFSS to 14.1% for HFSS + Met, *p* = 0.0310) and sensory (from 40.8% for HFSS to 14.6% for HFSS + Met, *p* = 0.0236) cortexes. The TNFα levels in brains quantified via ELISA were consistent with the microglial phenotype switching presented in Figures 3 and 4 and confirmed the pro‐inflammatory effects of HFSS and the anti‐inflammatory potential of Met (Figure S1).

Morphologically, the microglia in the HFSS group indicated severe hyperactivation, as evidenced by more microglia with large cell bodies (Figure 3A e‐h, q‐t; Figure 4A e‐h, q‐t) compared with the ND group (Figure 3A a‐d, m‐p, Figure 4A a‐d, m‐p). There were fewer Iba‐1^+^ microglia with large cell bodies in cerebral cortexes of Met + HFSS‐treated mice (Figure 3A i‐l, u‐x; Figure 4A i‐l, u‐x) than of HFSS‐treated mice (Figure 3A e‐h, q‐t; Figure 4A e‐h, q‐t). These results suggest that HFSS increases microglia reactivity, whereas Met tends to attenuate this increase.^65^

### 3.5 HFSS‐increased accumulation of phosphorylated tau protein (pTau) in the motor and sensory cortexes

**FIGURE 5:**
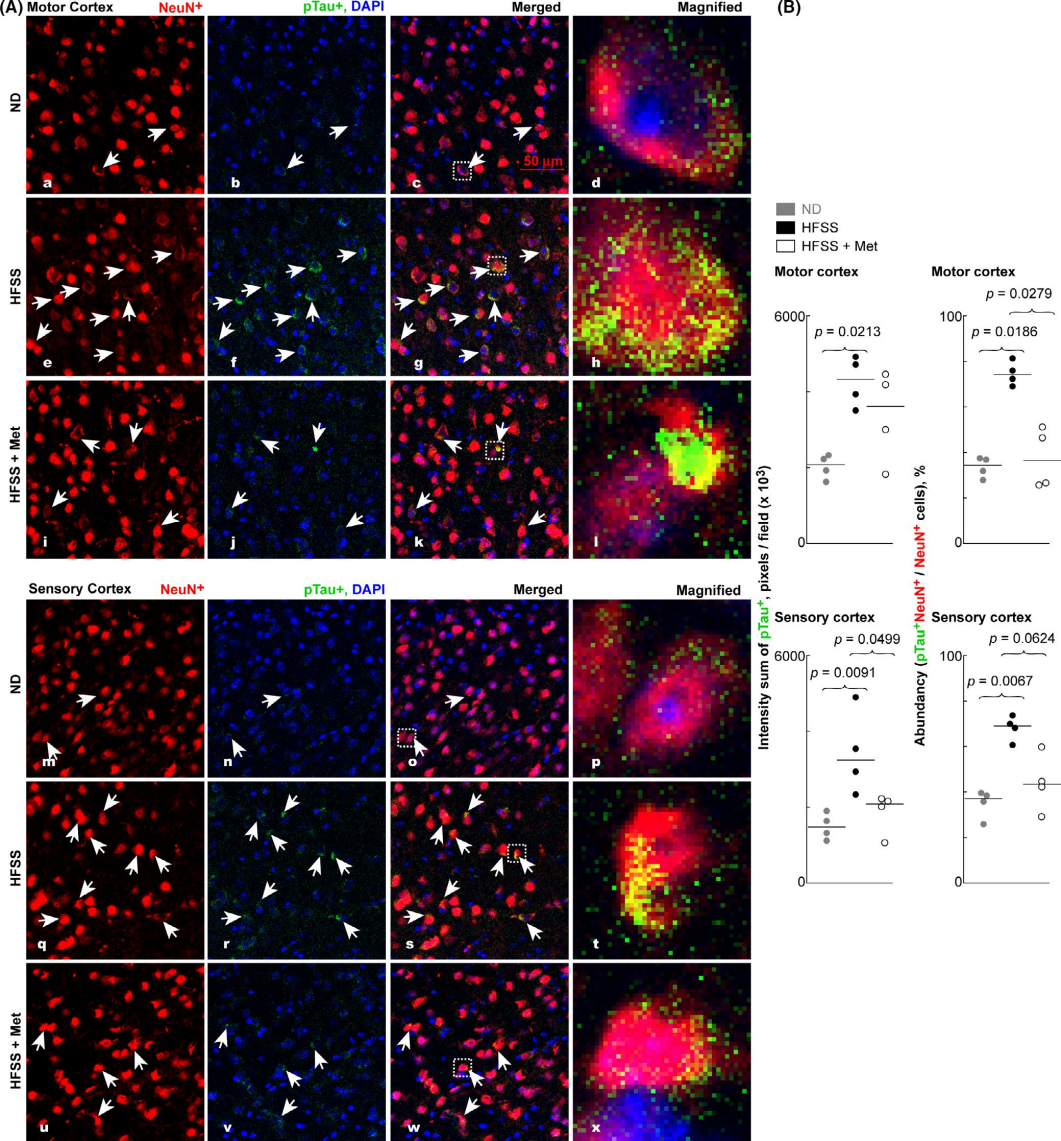
HFSS diet augmented pTau abundance in the motor and sensory cortexes of mice during aging, whereas some of Met treatment insignificantly curbed this HFSS effect. A, Microimages of pTau and NeuN in motor and sensory cortexes in brain cryosections. B, left: intensity sum of pTau^+^ green pixels per microscope field representing pTau levels in the brain cortexes; right: abundancy of pTau^+^NeuN^+^ / NeuN^+^cells, % per microscope field in the cortexes of B6 mice. The magnified images at the farthest right column of “A” show the typical expression and localization of pTau and NeuN. Data are presented as dots and lines (medians). *p* ≤ 0.05 is significant (*n* = 4 mice). Scale bar =50 µm

Tau and its phosphorylated form, pTau, are mainly involved in the stabilization of microtubule structure and the assembly of tubulin into microtubules. Overexpression of pTau is a hallmark of AD.^66^, ^67^, ^68^ Co‐staining of mouse brain sections with NeuN antibody and pSer199/202 pTau antibody revealed some pTau proteins along NeuN^+^ nuclei in the motor and sensory cortexes with more or less co‐localization (yellow color) of pTau and NeuN (Figure 5A). The pTau that co‐localized with NeuN (pTau^+^NeuN^+^, yellow spots) was very likely present in the neuronal soma. The ND‐fed aging mice showed visible pTau (green) accumulation in the motor and sensory cortexes (Figure 5A a‐d, m‐p). The pTau expression level increased profoundly in the HFSS‐fed mice, indicating the harmful effects of this diet on the motor and sensory cortexes (Figure 5A e‐h and q‐t, and B). Analysis of pTau revealed a significantly larger intensity sum of pTau^+^ pixels (green) per microscope field in the HFSS‐fed than in the ND‐fed mice in the motor cortex (Figure 5B upper, *p* = 0.0213) and sensory cortex (Figure 5B lower, *p* = 0.0091). Met treatment showed the trend of curbing HFSS‐augment of pTau^+^ levels in both motor (*p* = 0.1404, insignificant) and sensory (*p* = 0.0499) cortexes (Figure 5B left). HFSS also increased the abundancy of pTau^+^ neurons (pTau^+^NeuN^+^, yellow), % among the NeuN^+^neurons in motor (*p* = 0.0186) and sensory (*p* = 0.0067) cortexes and Met attenuated such HFSS‐linked increase of pTau^+^NeuN^+^ neuronal abundancy in motor (*p* = 0.0279) and sensory (*p* = 0.0624) cortexes (Figure 5B right).

### 3.6 Aβ deposition along the Col‐IV‐labeled blood vessels in motor and sensory cortex

**FIGURE 6:**
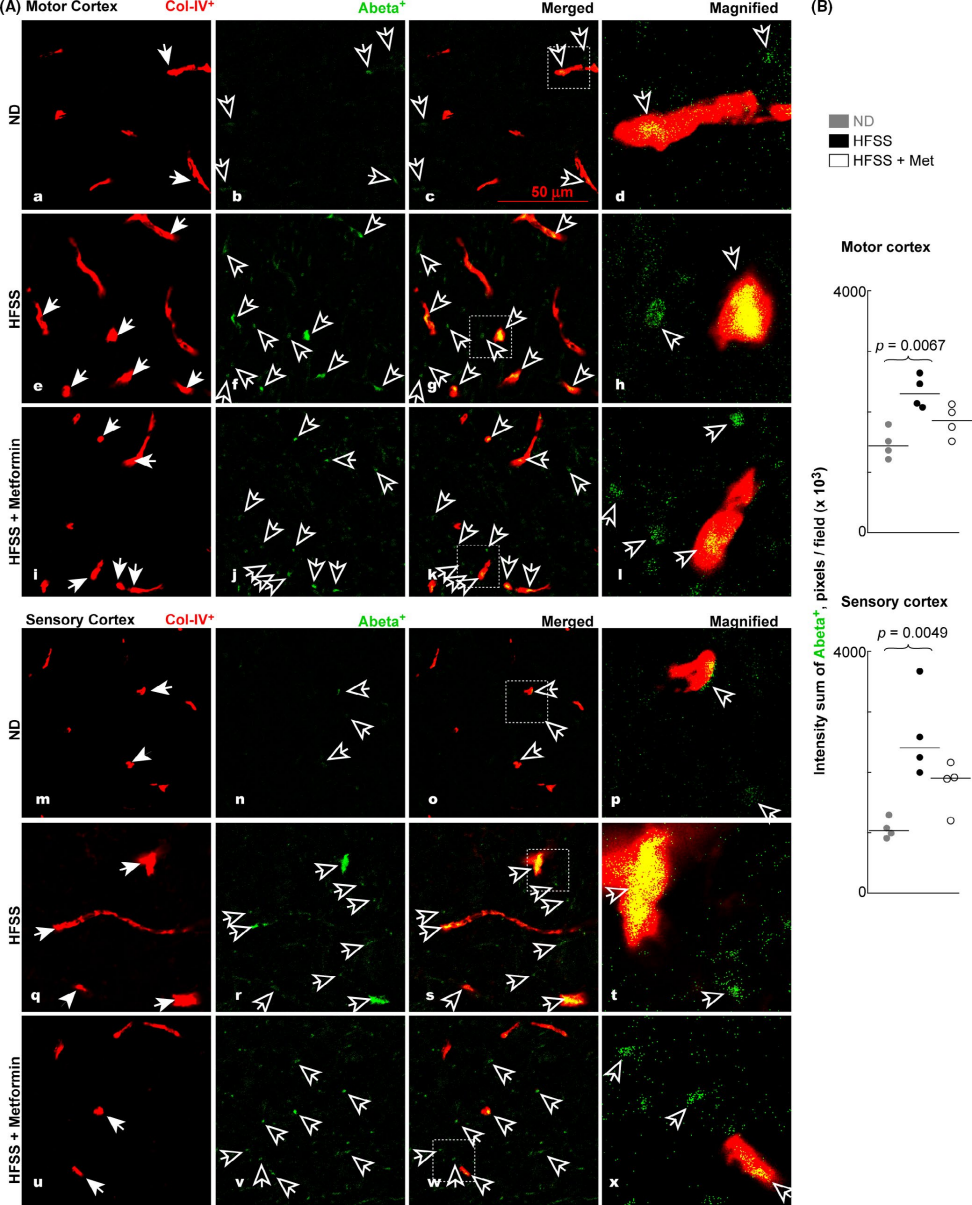
HFSS consumption induced Aβ accumulation in the vicinity of Col‐IV^+^ blood vessels in both motor and sensory cortexes of mice during aging, whereas Met treatment tended to curb this induction. A, Typical microimages of Aβ and Col‐IV in the cortexes in the brain cryosections of B6 mice. B, Intensity sum of Aβ^+^ pixels per microscopic field representing the densities of Aβ deposition. Solid arrows: blood vessels; hollow arrows: Aβ deposits. Data are presented as dots and lines (medians). *p* ≤ 0.05 is significant (*n* = 4 mice). Scale bar =50 µm

β‐amyloid (Aβ) deposition along neurovascular units is another characteristic of AD, in addition to the excessive expression of pTau.^66^, ^67^, ^68^ The co‐localization of some Aβ (green) and Col‐VI^+^ blood vessels (red) has been revealed as yellow regions in the brain cortexes (Figure 6), indicating Aβ deposition in/on blood vessels of the cortexes. The HFSS‐fed mice had increased Aβ deposition along (in or on) the Col‐IV‐labeled (Aβ^+^Col‐IV^+^) blood vessels in cortexes compared with ND‐fed mice (Figure 6A e‐h, q‐t vs. a‐d, m‐p). Interestingly, Met treatment reduced the Aβ deposition along the col‐IV labeled blood vessels in cortexes in the HFSS + Met group than in the HFSS‐fed group (Figure 6A i‐l, u‐x vs. e‐h, q‐t).

Quantitative analyses confirmed that the median of the Aβ^+^ pixel intensity sum per microscopic field in the motor cortexes was larger in the HFSS‐fed group than in the ND‐fed (*p* = 0.0067) or HFSS + Met (*p* = 0.1167) group (Figure 6B upper‐left). A similar pattern for this was observed in the sensory cortexes of the HFSS‐fed group compared with the ND‐fed (*p* = 0.0049) or HFSS + Met (*p* = 0.1413) group (Figure 6B lower‐left). No difference was observed in the Col‐IV^+^ intensity sum per microscopic field between groups (Figure 6A, quantitative data not shown).

Our statistical analysis indicated that the abundancy of M2‐like Arg‐1^+^Iba‐1^+^ microglia over total Iba‐1^+^ microglia (Figure 3) is negatively correlated with the level of Aβ (Figure 6) or pTau or portion of pTau^+^NeuN^+^/NeuN^+^ cells (Figure 5) in the mouse brain cortexes of HFSS, HFSS + Met, and ND groups [Aβ (*r* = −0.7193, *p* = 0.0106), pTau (*r* = −0.5088, *p* = 0.0936), and pTau^+^NeuN^+^/NeuN^+^ cells (*r* = −0.7614, *p* = 0.055) for the motor cortex; Aβ (*r* = −0.6725, *p* = 0.0202), pTau (*r* = −0.7636, *p* = 0.0055), and pTau^+^NeuN^+^/NeuN^+^ cells (*r* = −0.7566, *p* = 0.0062) for the sensory cortex]. In contrast, the abundancy of M1‐like iNOs^+^Iba‐1^+^ microglia over total Iba‐1^+^ microglia in the microscopic field (Figure 4) is positively correlated with the level of Aβ or pTau or the abundancy of pTau^+^NeuN^+^/NeuN^+^ cells in the brain cortexes [Aβ (*r* = 0.5874, *p* = 0.0489), pTau (*r* = 0.6783, *p* = 0.0185), and pTau^+^NeuN^+^/NeuN^+^ cells (*r* = 0.6294, *p* = 0.0323) for the motor cortex; Aβ (*r* = 0.5895, *p* = 0.0457), pTau (*r* = 0.6316, *p* = 0.0299), and pTau^+^NeuN^+^/NeuN^+^ cells (*r* = 0.6491, *p* = 0.0257) for the sensory cortex].

## 4 DISCUSSION

This study demonstrates that HFSS fosters neurological dysfunctions in the aged mice on cerebral cortexes in motor‐muscular strength and sensory behavioral functions, and neurodegenerative and microglial changes during aging. Our focus on HFSS has arisen from findings that Western diets having a single or double high feature of fat, sugar, and salt promulgate some neurodegeneration‐related characteristics.^4^, ^18^, ^37^, ^38^, ^39^, ^49^, ^50^ Some of these effects are more or less visible in rodents fed the diet for 2–8 months, depending on the exact diet, animal strain, and age.^4^, ^69^, ^70^, ^71^ However, the real‐world Western diets are usually high in all three components (fat, sugar, and salt) and are not well studied. This report has initially addressed the knowledge gap on the potential consequences of a Western diet combining HFSS on motor and sensory cortexes and related neurological outcomes. It also has provided a unique protocol of 2 months feeding, a relatively short duration compared to other similar studies in the field. Aging impairs the above functions and relevant cells and molecules,^72^, ^73^, ^74^, ^75^ thereby contributing to the outcomes observed in these old mice as they age from 20 to 22 months of age. Notably, this HFSS diet did not result in a significant difference in body weights (Figure S2) or blood glucose compared to the ND diet, suggesting that obesity or diabetes could not account for the neurodegenerative effects of HFSS. Our observations of 20–22 months‐old B6 mice paralleled observation of a previous report showing that 10 weeks of a high‐fat diet did not cause a significant change in the body weights of 5xFAD mice, but accelerated the pathogenesis of Alzheimer's‐like disease.^37^

We observed marked deficiencies in motor‐muscular grip strength and in heat sensitivities in HFSS‐fed compared with ND‐fed aged mice. Although our behavior studies are preliminary because of the small sample size in each group, the results are suggestive of significant effects that warrant future studies. The alterations in motor‐muscular grip strength and sensory abilities have been explained by neurotransmission modifications and cerebral atrophy in the motor and sensory cortexes.^73^, ^76^, ^77^, ^78^ These changes are likely to affect the cortico‐cortical and corticospinal connectivity.

We also observed lower densities of neurons marked by NeuN in the hemisphere of HFSS‐fed mice than ND‐fed mice, although the difference was not significant (*p* = 0.1302). Previous studies have shown neuronal death following the consumption of a high‐fat diet.^70^, ^79^ By contrast, another recent study reported that consumption of a high salt diet does not cause any neuronal death.^4^ Our study used HFSS, the combination of high fat, sugar, and salt common in Western diets, with the assumption that these components together would have a collective effect that could reduce motor‐muscular grip strength and sensory behavior in aged mice. These data suggest that future studies should quantitatively delineate the effects of HFSS on neuronal distribution in motor and sensory cortexes using larger sample sizes.

Our results showed that Met attenuated the above detrimental effects of HFSS diet. Our Met treatment offset some HFSS‐induced defects of cortex‐related behavior and neural cells in aging mice (Figures 1, 2, 3, 4, 5, 6), supporting a neuroprotective effect of Met during HFSS consumption and aging‐related neurodegeneration. The beneficial actions of Met are known to extend beyond diabetes management, since Met is also used in the treatment of various neurodegenerative disorders,^80^, ^81^, ^82^, ^83^, ^84^ motor dysfunctions,^85^ aging,^29^ and inflammatory signaling.^86^, ^87^, ^88^

Previous studies reported more microglia with a reactive morphology (large cell bodies) from the cerebral cortexes of aged mice fed ND than in young adult mice fed ND.^89^, ^90^ The HFSS diet resulted in even more microglia with the reactive morphology in the aged B6 mice compared with mice fed ND, consistent with increased neuroinflammation.^65^ This observation is also in accordance with a new genome‐wide transcriptomic study that indicated heightened chronic inflammation in aged microglia. A significant increase was also noted for the inflammatory iNOs^+^ microglia and a decrease in the anti‐inflammatory Arg‐1^+^ microglia in the cortexes, as well as an elevation of TNFα levels in brains of the HFSS‐fed aged mice compared with the ND‐fed aged mice (Figures 3,4, Figure S1). A high‐fat diet increases oxidative stress in both serum and tissues throughout the body,^7^, ^11^, ^12^ consequently increases the influx of oxidative stress radicals across the blood‐brain barrier into the brain.^8^, ^71^ Excessive oxidative radicals and inflammatory mediators, including TNFα,^8^ are therefore likely to play a role in neurodegeneration, in the inflammatory activation of microglia, and in the increased expression of iNOs observed in cortexes of HFSS‐fed aged mice, as observed in the present study (Figure 4). In cortexes of the HFSS‐fed aged mice, Met treatment not only reduced the levels of inflammatory TNFα and iNOs^+^‐microglia, but also increased the level of anti‐inflammatory Arg‐1^+^ microglia, demonstrating an immunomodulating action of Met on microglia cells (Figure S1, Figures 3,4). Moreover, aging impaired the regenerative functions of microglia,^89^, ^90^ which is likely to interplay with HFSS in neurodegeneration and invites future study to address the novel interaction.

HFSS augmented Aβ clustering along blood vessels as well as Aβ and pTau accumulation in the cortexes (Figures 5 and 6). This is consistent with the HFSS‐induced reduction of Arg‐1^+^ microglia and surge of inflammatory iNOs^+^ microglia (Figures 3,4) because Inflammatory phenotype of microglia has lower ability in phagocytosis to clean or reduce excessive Aβ and pTau compared with inflammation‐resolving Arg‐1^+^ microglia.^32^, ^33^, ^34^, ^35^, ^36^ In our results from ND, HFSS, and HFSS + Met groups, there is a negative correlation between the M2‐like Arg‐1^+^Iba‐1^+^ microglial abundancy and the level of Aβ or pTau or pTau^+^NeuN^+^cell abundancy in brain cortexes, whereas the correlation between M1‐like iNOs^+^Iba‐1^+^ microglia abundancy and the level of Aβ or pTau or pTau^+^NeuN^+^cell abundancy is positive. Moreover, Aβ and pTau can promote inflammation, thus switch microglia to M1 type and foster neuroinflammation, neurodegeneration, and incidence of AD.^32^, ^33^ Aβ deposition in the vicinity of a blood vessel wall can lead to vascular rupture and intracerebral hemorrhage.^40^, ^41^ Our results are consistent with the knowledge that M2 microglia are more effective than M1 microglia in the phagocytosis of extracellular Aβ and Tau/pTau, and that extracellular Aβ and pTau are pro‐inflammatory, consequently may promote the switch from M2 to M1 microglia.^32^, ^33^, ^34^, ^35^, ^36^, ^91^

The microimages acquired for co‐staining of NeuN and pTau (pSer199/202) revealed that HFSS also increased the pTau level in cortexes and in NeuN^+^ neuronal somas (pTau^+^NeuN^+^) of these cortexes.^32^, ^36^, ^66^, ^67^, ^68^, ^92^ These findings indicate that long‐term consumption of a combined HFSS diet might exacerbate AD susceptibility and progression in the aged brain. Moreover, Met tended to curb the accumulation of Aβ along blood vessels and pTau, suggesting neuroprotection by Met against AD‐like changes and the aging process.^21^, ^93^, ^94^, ^95^ Further experiments are warranted to determine whether Met is more effective with longer treatment or at higher doses. Whether Met prevents or promotes the removal of Aβ and pTau accumulation also warrants future investigation.

This exploratory study tested male mice only. Numerous studies suggest the existence of sex differences in brain metabolism,^96^ microglial activation and neuroinflammation,^97^, ^98^ and vulnerability to brain injury,^99^, ^100^ which are involved in the AD neurodegeneration. Thus, this study needs to be also conducted on female mice in the future to test the prediction of sex differences on the effects of HFSS diet and Met and involved mechanisms.

## 5 CONCLUSIONS

Our data showed that aging male B6 mice fed a HFSS diet for 2 months developed motor‐muscular and sensory defects and displayed an inflammatory switch of microglial phenotypes and TNFα production. In addition, it demonstrates an increased burden of Aβ and pTau in the motor and sensory cortexes. This study is the first exploration, to the best of our knowledge, of the connection between a Western diet combining all components of HFSS and the early events leading to impaired motor and sensory functions in the aged. The HFSS‐induced pathogenesis disclosed here provides an effective window into mechanisms involved, while targeting potential new therapeutic avenues. The ameliorating neuroprotection of Met in HFSS‐induced pathogenesis is promising and requires future investigation.^101^, ^102^, ^103^, ^104^ Additional exploration of genetically traceable motor and sensory circuits will further augment our understanding of HFSS‐induced defects at both the circuit and network levels. Relative to the lifelong dietary style of some humans, our chronic HFSS treatment could be still too short; therefore, a longer HFSS duration deserves future investigation. HFSS, aging, and Met could also affect vascular structures and blood pressure^19^, ^105^; thus, this possibility should be studied. We recommend a more explorative paradigm in this area that examines various aspects, such as longer time for the pathogenesis, different Met dosages, the comparison of HFSS versus a Western diet with single or double high levels of its three components at different ages to identify the most critical factors leading to neurological defects, and the effects of Met at different life stages to advance the therapeutic approaches to curb pathogenesis. We anticipate that the findings of this study could lead to improved tools for effective intervention and a better understanding of chronic neural diseases, including AD.

## CONFLICTS OF INTEREST

The authors declare that they have no conflict of interest.

## AUTHOR CONTRIBUTIONS

SH involved in conceiving the entire project, conceptualization, experimental design, data curation, validation, writing, supervision, and funding acquisition. AN and YL involved in data curation, formal analysis, validation, and writing. HP: experimental design, formal analysis, data validation, and writing. QAVD involved in data curation and validation. NBP and CAV involved in data curation. NGB involved in conceptualization, data interpretation, validation, writing, reviewing and editing, and resources.

## Supporting information

Supplementary Material

Supplementary Material

## Data Availability

The data of this report are available from the corresponding author upon reasonable request.

## References

[1] Barnes DE , Yaffe K . The projected effect of risk factor reduction on Alzheimer's disease prevalence. Lancet Neurol. 2011;10(9):819‐828. 21775213 10.1016/S1474-4422(11)70072-2 PMC3647614

[2] Sherzai A , Heim LT , Boothby C , Sherzai AD . Stroke, food groups, and dietary patterns: a systematic review. Nutr Rev. 2012;70(8):423‐435. 22835136 10.1111/j.1753-4887.2012.00490.x

[3] Theriault P , ElAli A , Rivest S . High fat diet exacerbates Alzheimer's disease‐related pathology in APPswe/PS1 mice. Oncotarget. 2016;7(42):67808‐67827. 27661129 10.18632/oncotarget.12179 PMC5356521

[4] Faraco G , Hochrainer K , Segarra SG , et al. Dietary salt promotes cognitive impairment through tau phosphorylation. Nature. 2019;574(7780):686‐690. 31645758 10.1038/s41586-019-1688-z PMC7380655

[5] Oikonomou E , Psaltopoulou T , Georgiopoulos G , et al. Western dietary pattern is associated with severe coronary artery disease. Angiology. 2018;69(4):339‐346. 28731359 10.1177/0003319717721603

[6] Santisteban MM , Iadecola C . Hypertension, dietary salt and cognitive impairment. J Cereb Blood Flow Metab. 2018;38(12):2112‐2128. 30295560 10.1177/0271678X18803374 PMC6282225

[7] Studzinski CM , Li F , Bruce‐Keller AJ , et al. Effects of short‐term western diet on cerebral oxidative stress and diabetes related factors in APP x PS1 knock‐in mice. J Neurochem. 2009;108(4):860‐866. 19046405 10.1111/j.1471-4159.2008.05798.x PMC2748316

[8] Hsu TM , Kanoski SE . Blood‐brain barrier disruption: mechanistic links between Western diet consumption and dementia. Front Aging Neurosci. 2014;6:88. 24847262 10.3389/fnagi.2014.00088 PMC4023063

[9] Hansson GK , Hermansson A . The immune system in atherosclerosis. Nat Immunol. 2011;12(3):204‐212. 21321594 10.1038/ni.2001

[10] Soehnlein O , Swirski FK . Hypercholesterolemia links hematopoiesis with atherosclerosis. Trends Endocrinol Metab. 2013;24(3):129‐136. 23228326 10.1016/j.tem.2012.10.008 PMC4302393

[11] Munoz A , Costa M . Nutritionally mediated oxidative stress and inflammation. Oxid Med Cell Longev. 2013;2013:610950. 23844276 10.1155/2013/610950 PMC3697417

[12] Tan BL , Norhaizan ME , Liew WP . Nutrients and oxidative stress: friend or foe? Oxid Med Cell Longev. 2018;2018:9719584. 29643982 10.1155/2018/9719584 PMC5831951

[13] Veniaminova E , Cespuglio R , Chernukha I , et al. Metabolic, molecular, and behavioral effects of western diet in serotonin transporter‐deficient mice: rescue by heterozygosity? Front Neurosci. 2020;14:24. 32132889 10.3389/fnins.2020.00024 PMC7041415

[14] Duque‐Guimaraes D , Ozanne S . Early nutrition and ageing: can we intervene? Biogerontology. 2017;18(6):893‐900. 28357523 10.1007/s10522-017-9691-y PMC5684303

[15] McDonald RB , Ramsey JJ . Honoring clive McCay and 75 years of calorie restriction research. J Nutr. 2010;140(7):1205‐1210. 20484554 10.3945/jn.110.122804 PMC2884327

[16] Ogden CL , Carroll MD , Kit BK , Flegal KM . Prevalence of childhood and adult obesity in the United States, 2011‐2012. JAMA. 2014;311(8):806‐814. 24570244 10.1001/jama.2014.732 PMC4770258

[17] Chugh G , Asghar M , Patki G , et al. A high‐salt diet further impairs age‐associated declines in cognitive, behavioral, and cardiovascular functions in male Fischer brown Norway rats. J Nutr. 2013;143(9):1406‐1413. 23864508 10.3945/jn.113.177980 PMC3743272

[18] Faraco G , Brea D , Garcia‐Bonilla L , et al. Dietary salt promotes neurovascular and cognitive dysfunction through a gut‐initiated TH17 response. Nat Neurosci. 2018;21(2):240‐249. 29335605 10.1038/s41593-017-0059-z PMC6207376

[19] Petersen JS , Andersen D , Muntzel MS , Diemer NH , Holstein‐Rathlou NH . Intracerebroventricular metformin attenuates salt‐induced hypertension in spontaneously hypertensive rats. Am J Hypertens. 2001;14(11 Pt 1):1116‐1122. 11724210 10.1016/s0895-7061(01)02220-8

[20] Brietzke SA . Oral antihyperglycemic treatment options for type 2 diabetes mellitus. Med Clin North Am. 2015;99(1):87‐106. 25456645 10.1016/j.mcna.2014.08.012

[21] Chen YY , Shen YC , Lai YJ , et al. Association between metformin and a lower risk of age‐related macular degeneration in patients with type 2 diabetes. J Ophthalmol. 2019;2019:1649156. 31781371 10.1155/2019/1649156 PMC6875398

[22] Perez‐Revuelta BI , Hettich MM , Ciociaro A , et al. Metformin lowers Ser‐129 phosphorylated alpha‐synuclein levels via mTOR‐dependent protein phosphatase 2A activation. Cell Death Dis. 2014;5:e1209. 24810045 10.1038/cddis.2014.175 PMC4047877

[23] Jiang T , Yu JT , Zhu XC , et al. Acute metformin preconditioning confers neuroprotection against focal cerebral ischaemia by pre‐activation of AMPK‐dependent autophagy. Br J Pharmacol. 2014;171(13):3146‐3157. 24611741 10.1111/bph.12655 PMC4080970

[24] Hsu CC , Wahlqvist ML , Lee MS , Tsai HN . Incidence of dementia is increased in type 2 diabetes and reduced by the use of sulfonylureas and metformin. J Alzheimers Dis. 2011;24(3):485‐493. 21297276 10.3233/JAD-2011-101524

[25] Ng TP , Feng L , Yap KB , Lee TS , Tan CH , Winblad B . Long‐term metformin usage and cognitive function among older adults with diabetes. J Alzheimers Dis. 2014;41(1):61‐68. 24577463 10.3233/JAD-131901

[26] Oliveira WH , Nunes AK , Franca ME , et al. Effects of metformin on inflammation and short‐term memory in streptozotocin‐induced diabetic mice. Brain Res. 2016;1644:149‐160. 27174003 10.1016/j.brainres.2016.05.013

[27] Vazquez‐Manrique RP , Farina F , Cambon K , et al. AMPK activation protects from neuronal dysfunction and vulnerability across nematode, cellular and mouse models of Huntington's disease. Hum Mol Genet. 2016;25(6):1043‐1058. 26681807 10.1093/hmg/ddv513 PMC4764188

[28] Femminella GD , Bencivenga L , Petraglia L , et al. Antidiabetic drugs in Alzheimer's disease: mechanisms of action and future perspectives. J Diabetes Res. 2017;2017:7420796. 28656154 10.1155/2017/7420796 PMC5471577

[29] Martin‐Montalvo A , Mercken EM , Mitchell SJ , et al. Metformin improves healthspan and lifespan in mice. Nat Commun. 2013;4:2192. 23900241 10.1038/ncomms3192 PMC3736576

[30] Fatt M , Hsu K , He L , et al. Metformin acts on two different molecular pathways to enhance adult neural precursor proliferation/self‐renewal and differentiation. Stem cell reports. 2015;5(6):988‐995. 26677765 10.1016/j.stemcr.2015.10.014 PMC4682208

[31] Alfaras I , Mitchell SJ , Mora H , et al. Health benefits of late‐onset metformin treatment every other week in mice. NPJ aging and mechanisms of disease. 2017;3:16. 29167747 10.1038/s41514-017-0018-7 PMC5696465

[32] Cherry JD , Olschowka JA , O'Banion MK . Neuroinflammation and M2 microglia: the good, the bad, and the inflamed. J Neuroinflammation. 2014;11:98. 24889886 10.1186/1742-2094-11-98 PMC4060849

[33] Lisi L , Ciotti GM , Braun D , et al. Expression of iNOS, CD163 and ARG‐1 taken as M1 and M2 markers of microglial polarization in human glioblastoma and the surrounding normal parenchyma. Neurosci Lett. 2017;645:106‐112. 28259657 10.1016/j.neulet.2017.02.076

[34] Yao PL , Zhuo S , Mei H , et al. Androgen alleviates neurotoxicity of beta‐amyloid peptide (Abeta) by promoting microglial clearance of abeta and inhibiting microglial inflammatory response to abeta. CNS Neurosci Ther. 2017;23(11):855‐865. 28941188 10.1111/cns.12757 PMC6492702

[35] Hu MY , Lin YY , Zhang BJ , Lu DL , Lu ZQ , Cai W . Update of inflammasome activation in microglia/macrophage in aging and aging‐related disease. CNS Neurosci Ther. 2019;25(12):1299‐1307. 31729181 10.1111/cns.13262 PMC6887669

[36] Zhang F , Zhong R , Li S , et al. Acute hypoxia induced an imbalanced M1/M2 activation of microglia through NF‐kappaB signaling in Alzheimer's disease mice and wild‐type littermates. Front Aging Neurosci. 2017;9:282. 28890695 10.3389/fnagi.2017.00282 PMC5574879

[37] Lin B , Hasegawa Y , Takane K , Koibuchi N , Cao C , Kim‐Mitsuyama S . High‐fat‐diet intake enhances cerebral amyloid angiopathy and cognitive impairment in a mouse model of Alzheimer's disease, independently of metabolic disorders. J Am Heart Assoc. 2016;5(6):e003154. 27412896 10.1161/JAHA.115.003154 PMC4937262

[38] Kothari V , Luo Y , Tornabene T , et al. High fat diet induces brain insulin resistance and cognitive impairment in mice. Biochim Biophys Acta Mol Basis Dis. 2017;1863(2):499‐508. 27771511 10.1016/j.bbadis.2016.10.006

[39] Fyfe I . High‐salt diet promotes Alzheimer disease‐like changes. Nat Rev Neurol. 2020;16(1):2‐3. 10.1038/s41582-019-0289-7 31712717

[40] Niwa K , Kazama K , Younkin SG , Carlson GA , Iadecola C . Alterations in cerebral blood flow and glucose utilization in mice overexpressing the amyloid precursor protein. Neurobiol Dis. 2002;9(1):61‐68. 11848685 10.1006/nbdi.2001.0460

[41] Smith EE , Greenberg SM . Beta‐amyloid, blood vessels, and brain function. Stroke. 2009;40(7):2601‐2606. 19443808 10.1161/STROKEAHA.108.536839 PMC2704252

[42] Siparsky PN , Kirkendall DT , Garrett WE Jr . Muscle changes in aging: understanding sarcopenia. Sports Health. 2014;6(1):36‐40. 24427440 10.1177/1941738113502296 PMC3874224

[43] Wickremaratchi MM , Llewelyn JG . Effects of ageing on touch. Postgrad Med J. 2006;82(967):301‐304. 16679466 10.1136/pgmj.2005.039651 PMC2563781

[44] Vanhooren V , Libert C . The mouse as a model organism in aging research: usefulness, pitfalls and possibilities. Ageing Res Rev. 2013;12(1):8‐21. 22543101 10.1016/j.arr.2012.03.010

[45] Ackert‐Bicknell CL , Anderson LC , Sheehan S , et al. Aging research using mouse models. Curr Protoc Mouse Biol. 2015;5(2):95‐133. 26069080 10.1002/9780470942390.mo140195 PMC4590775

[46] Dutta S , Sengupta P . Men and mice: relating their ages. Life Sci. 2016;152:244‐248. 26596563 10.1016/j.lfs.2015.10.025

[47] Nomura T , Li XH , Ogata H , et al. Suppressive effects of continuous low‐dose‐rate gamma irradiation on diabetic nephropathy in type II diabetes mellitus model mice. Radiat Res. 2011;176(3):356‐365. 21718105 10.1667/rr2559.1

[48] Percie du Sert N , Hurst V , Ahluwalia A , et al. The ARRIVE guidelines 2.0: updated guidelines for reporting animal research. J Cereb Blood Flow Metab. 2020;40(9):1769‐1777. 32663096 10.1177/0271678X20943823 PMC7430098

[49] Petrov D , Pedros I , Artiach G , et al. High‐fat diet‐induced deregulation of hippocampal insulin signaling and mitochondrial homeostasis deficiences contribute to Alzheimer disease pathology in rodents. Biochim Biophys Acta. 2015;1852(9):1687‐1699. 26003667 10.1016/j.bbadis.2015.05.004

[50] Davidson TL , Jones S , Roy M , Stevenson RJ . The cognitive control of eating and body weight: it's more than what you "think". Front Psychol. 2019;10:62. 30814963 10.3389/fpsyg.2019.00062 PMC6381074

[51] Deacon RM . Measuring the strength of mice. J Vis Exp. 2013;76:2610. 10.3791/2610 PMC3725666 23770643

[52] Deuis JR , Dvorakova LS , Vetter I . Methods used to evaluate pain behaviors in rodents. Front Mol Neurosci. 2017;10:284. 28932184 10.3389/fnmol.2017.00284 PMC5592204

[53] Marcheselli VL , Hong S , Lukiw WJ , et al. Novel docosanoids inhibit brain ischemia‐reperfusion‐mediated leukocyte infiltration and pro‐inflammatory gene expression. J Biol Chem. 2003;278(44):43807‐43817. 12923200 10.1074/jbc.M305841200

[54] Nagayach A , Patro N , Patro I . Experimentally induced diabetes causes glial activation, glutamate toxicity and cellular damage leading to changes in motor function. Front Cell Neurosci. 2014;8:355. 25400546 10.3389/fncel.2014.00355 PMC4215794

[55] Nagayach A , Patro N , Patro I . Astrocytic and microglial response in experimentally induced diabetic rat brain. Metab Brain Dis. 2014;29(3):747‐761. 24833555 10.1007/s11011-014-9562-z

[56] Hong S , Alapure BV , Lu Y , Tian H , Wang Q . 12/15‐Lipoxygenase deficiency reduces densities of mesenchymal stem cells in the dermis of wounded and unwounded skin. Br J Dermatol. 2014;171(1):30‐38. 24593251 10.1111/bjd.12899 PMC4114990

[57] Hong S , Alapure BV , Lu Y , Tian H , Wang Q . Immunohistological localization of endogenous unlabeled stem cells in wounded skin. J Histochem Cytochem. 2014;62(4):276‐285. 24399040 10.1369/0022155414520710 PMC3966289

[58] Alapure BV , Lu Y , He M , et al. Accelerate healing of severe burn wounds by mouse bone marrow mesenchymal stem cell‐seeded biodegradable hydrogel scaffold synthesized from arginine‐based poly(ester amide) and chitosan. Stem Cells Dev. 2018;27(23):1605‐1620. 30215325 10.1089/scd.2018.0106 PMC6276600

[59] Nagayach A , Singh A , Geller AI . Delivery of different genes into presynaptic and postsynaptic neocortical neurons connected by a BDNF‐TrkB synapse. Brain Res. 2019;1712:16‐24. 30710509 10.1016/j.brainres.2019.01.038

[60] Paxinos G , Franklin K . Paxinos and Franklin's the Mouse Brain in Stereotaxic Coordinates, 2nd ed. Academic Press; 2001.

[61] Jin K , Peel AL , Mao XO , et al. Increased hippocampal neurogenesis in Alzheimer's disease. Proc Natl Acad Sci USA. 2004;101(1):343‐347. 14660786 10.1073/pnas.2634794100 PMC314187

[62] Mao S , Xiong G , Zhang L , et al. Verification of the cross immunoreactivity of A60, a mouse monoclonal antibody against neuronal nuclear protein. Front Neuroanat. 2016;10:54. 27242450 10.3389/fnana.2016.00054 PMC4865646

[63] Myers JL , Well AD . Research Design and Statistical Analysis. 2nd ed. Lawrence Erlbaum Associates; 2003.

[64] Conover WJ . Practical Nonparametric Statistics. 3rd ed. Johns Wiley & Sons; 1999.

[65] Eggen BJ , Raj D , Hanisch UK , Boddeke HW . Microglial phenotype and adaptation. J Neuroimmune Pharmacol. 2013;8(4):807‐823. 23881706 10.1007/s11481-013-9490-4

[66] Xia Y , Prokop S , Gorion KM , et al. Tau Ser208 phosphorylation promotes aggregation and reveals neuropathologic diversity in Alzheimer's disease and other tauopathies. Acta Neuropathol Commun. 2020;8(1):88. 32571418 10.1186/s40478-020-00967-w PMC7310041

[67] Goedert M , Spillantini MG , Jakes R , Rutherford D , Crowther RA . Multiple isoforms of human microtubule‐associated protein tau: sequences and localization in neurofibrillary tangles of Alzheimer's disease. Neuron. 1989;3(4):519‐526. 2484340 10.1016/0896-6273(89)90210-9

[68] Brunello CA , Merezhko M , Uronen RL , Huttunen HJ . Mechanisms of secretion and spreading of pathological tau protein. Cell Mol life Sci. 2020;77(9):1721‐1744. 31667556 10.1007/s00018-019-03349-1 PMC7190606

[69] Carvalho C , Cardoso S , Correia SC , et al. Metabolic alterations induced by sucrose intake and Alzheimer's disease promote similar brain mitochondrial abnormalities. Diabetes. 2012;61(5):1234‐1242. 22427376 10.2337/db11-1186 PMC3331754

[70] Graham LC , Harder JM , Soto I , de Vries WN , John SW , Howell GR . Chronic consumption of a western diet induces robust glial activation in aging mice and in a mouse model of Alzheimer's disease. Sci Rep. 2016;6:21568. 26888450 10.1038/srep21568 PMC4757836

[71] Rutkowsky JM , Lee LL , Puchowicz M , et al. Reduced cognitive function, increased blood‐brain‐barrier transport and inflammatory responses, and altered brain metabolites in LDLr ‐/‐and C57BL/6 mice fed a western diet. PLoS One. 2018;13(2):e0191909. 29444171 10.1371/journal.pone.0191909 PMC5812615

[72] Stevens JC , Cruz LA , Marks LE , Lakatos S . A multimodal assessment of sensory thresholds in aging. J Gerontol B Psychol Sci Soc Sci. 1998;53(4):P263‐272. 9679518 10.1093/geronb/53b.4.p263

[73] Clark BC , Taylor JL . Age‐related changes in motor cortical properties and voluntary activation of skeletal muscle. Curr Aging Sci. 2011;4(3):192‐199. 21529329 10.2174/1874609811104030192 PMC3184350

[74] Schubert CR , Fischer ME , Pinto AA , et al. Sensory Impairments and risk of mortality in older adults. J Gerontol A Biol Sci Med Sci. 2017;72(5):710‐715. 26946102 10.1093/gerona/glw036 PMC5861964

[75] Cavazzana A , Rohrborn A , Garthus‐Niegel S , Larsson M , Hummel T , Croy I . Sensory‐specific impairment among older people. an investigation using both sensory thresholds and subjective measures across the five senses. PLoS One. 2018;13(8):e0202969. 30148857 10.1371/journal.pone.0202969 PMC6110574

[76] Rantanen T , Guralnik JM , Foley D , et al. Midlife hand grip strength as a predictor of old age disability. JAMA. 1999;281(6):558‐560. 10022113 10.1001/jama.281.6.558

[77] Salat DH , Buckner RL , Snyder AZ , et al. Thinning of the cerebral cortex in aging. Cereb Cortex. 2004;14(7):721‐730. 15054051 10.1093/cercor/bhh032

[78] Ward NS , Swayne OB , Newton JM . Age‐dependent changes in the neural correlates of force modulation: an fMRI study. Neurobiol Aging. 2008;29(9):1434‐1446. 17566608 10.1016/j.neurobiolaging.2007.04.017 PMC2568861

[79] Khan M , Rutten BPF , Kim MO . MST1 regulates neuronal cell death via JNK/Casp3 signaling pathway in HFD mouse brain and HT22 cells. Int J Mol Sci. 2019;20(10):2504. 10.3390/ijms20102504 PMC6566356 31117242

[80] Guo M , Mi J , Jiang QM , et al. Metformin may produce antidepressant effects through improvement of cognitive function among depressed patients with diabetes mellitus. Clin Exp Pharmacol Physiol. 2014;41(9):650‐656. 24862430 10.1111/1440-1681.12265

[81] Thangthaeng N , Rutledge M , Wong JM , Vann PH , Forster MJ , Sumien N . Metformin impairs spatial memory and visual acuity in old male mice. Aging Dis. 2017;8(1):17‐30. 28203479 10.14336/AD.2016.1010 PMC5287385

[82] Li W , Chaudhari K , Shetty R , et al. Metformin alters locomotor and cognitive function and brain metabolism in normoglycemic mice. Aging Dis. 2019;10(5):949‐963. 31595194 10.14336/AD.2019.0120 PMC6764722

[83] DiTacchio KA , Heinemann SF , Dziewczapolski G . Metformin treatment alters memory function in a mouse model of Alzheimer's disease. J Alzheimers Dis. 2015;44(1):43‐48. 25190626 10.3233/JAD-141332 PMC9057391

[84] Farr SA , Roesler E , Niehoff ML , Roby DA , McKee A , Morley JE . Metformin improves learning and memory in the SAMP8 mouse model of Alzheimer's disease. J Alzheimers Dis. 2019;68(4):1699‐1710. 30958364 10.3233/JAD-181240

[85] Allard JS , Perez EJ , Fukui K , Carpenter P , Ingram DK , de Cabo R . Prolonged metformin treatment leads to reduced transcription of Nrf2 and neurotrophic factors without cognitive impairment in older C57BL/6J mice. Behav Brain Res. 2016;301:1‐9. 26698400 10.1016/j.bbr.2015.12.012 PMC4823003

[86] Isoda K , Young JL , Zirlik A , et al. Metformin inhibits proinflammatory responses and nuclear factor‐kappaB in human vascular wall cells. Arterioscler Thromb Vasc Biol. 2006;26(3):611‐617. 16385087 10.1161/01.ATV.0000201938.78044.75

[87] Gu J , Ye S , Wang S , Sun W , Hu Y . Metformin inhibits nuclear factor‐kappaB activation and inflammatory cytokines expression induced by high glucose via adenosine monophosphate‐activated protein kinase activation in rat glomerular mesangial cells in vitro. Chin Med J (Engl). 2014;127(9):1755‐1760. 24791887

[88] Cameron AR , Morrison VL , Levin D , et al. Anti‐inflammatory effects of metformin irrespective of diabetes status. Circ Res. 2016;119(5):652‐665. 27418629 10.1161/CIRCRESAHA.116.308445 PMC4990459

[89] Shi L , Rocha M , Zhang W , et al. Genome‐wide transcriptomic analysis of microglia reveals impaired responses in aged mice after cerebral ischemia. J Cereb Blood Flow Metab. 2020;40(Suppl. 1):S49‐S66. 32438860 10.1177/0271678X20925655 PMC7687039

[90] Jiang L , Mu H , Xu F , et al. Transcriptomic and functional studies reveal undermined chemotactic and angiostimulatory properties of aged microglia during stroke recovery. J Cereb Blood Flow Metab. 2020;40(Suppl. 1):S81‐S97. 32065074 10.1177/0271678X20902542 PMC7687033

[91] Perea JR , Llorens‐Martin M , Avila J , Bolos M . The role of microglia in the spread of tau: relevance for tauopathies. Front Cell Neurosci. 2018;12:172. 30042659 10.3389/fncel.2018.00172 PMC6048186

[92] Brandt R , Leger J , Lee G . Interaction of tau with the neural plasma membrane mediated by tau's amino‐terminal projection domain. J Cell Biol. 1995;131(5):1327‐1340. 8522593 10.1083/jcb.131.5.1327 PMC2120645

[93] Anisimov VN , Berstein LM , Egormin PA , et al. Effect of metformin on life span and on the development of spontaneous mammary tumors in HER‐2/neu transgenic mice. Exp Gerontol. 2005;40(8–9):685‐693. 16125352 10.1016/j.exger.2005.07.007

[94] Kickstein E , Krauss S , Thornhill P , et al. Biguanide metformin acts on tau phosphorylation via mTOR/protein phosphatase 2A (PP2A) signaling. Proc Natl Acad Sci U S A. 2010;107(50):21830‐21835. 21098287 10.1073/pnas.0912793107 PMC3003072

[95] Li J , Deng J , Sheng W , Zuo Z . Metformin attenuates Alzheimer's disease‐like neuropathology in obese, leptin‐resistant mice. Pharmacol Biochem Behav. 2012;101(4):564‐574. 22425595 10.1016/j.pbb.2012.03.002 PMC3327803

[96] Vandekar SN , Shou H , Satterthwaite TD , et al. Sex differences in estimated brain metabolism in relation to body growth through adolescence. J Cereb Blood Flow Metab. 2019;39(3):524‐535. 10.1177/0271678X17737692 PMC6421255 29072856

[97] Li LZ , Huang YY , Yang ZH , Zhang SJ , Han ZP , Luo YM . Potential microglia‐based interventions for stroke. CNS Neurosci Ther. 2020;26(3):288‐296. 32064759 10.1111/cns.13291 PMC7052807

[98] Kerr N , Dietrich DW , Bramlett HM , Raval AP . Sexually dimorphic microglia and ischemic stroke. CNS Neurosci Ther. 2019;25(12):1308‐1317. 31747126 10.1111/cns.13267 PMC6887716

[99] Zhao L , Mulligan MK , Nowak TS Jr . Substrain‐ and sex‐dependent differences in stroke vulnerability in C57BL/6 mice. J Cereb Blood Flow Metab. 2019;39(3):426‐438. 29260927 10.1177/0271678X17746174 PMC6421252

[100] Bushnell CD , Chaturvedi S , Gage KR , et al. Sex differences in stroke: challenges and opportunities. J Cereb Blood Flow Metab. 2018;38(12):2179‐2191. 30114967 10.1177/0271678X18793324 PMC6282222

[101] Tarkowski E , Liljeroth AM , Nilsson A , Minthon L , Blennow K . Decreased levels of intrathecal interleukin 1 receptor antagonist in Alzheimer's disease. Dement Geriatr Cogn Disord. 2001;12(5):314‐317. 11455132 10.1159/000051276

[102] Serhan CN , Yang R , Martinod K , et al. Maresins: novel macrophage mediators with potent antiinflammatory and proresolving actions. J Exp Med. 2009;206(1):15‐23. 19103881 10.1084/jem.20081880 PMC2626672

[103] Schwab JM , Chiang N , Arita M , Serhan CN . Resolvin E1 and protectin D1 activate inflammation‐resolution programmes. Nature. 2007;447(7146):869‐874. 17568749 10.1038/nature05877 PMC2757086

[104] Zhu M , Wang X , Hjorth E , et al. Pro‐resolving lipid mediators improve neuronal survival and increase abeta42 phagocytosis. Mol Neurobiol. 2016;53(4):2733‐2749. 26650044 10.1007/s12035-015-9544-0 PMC4824659

[105] Soukas AA , Hao H , Wu L . Metformin as anti‐aging therapy: is it for everyone? Trends Endocrinol Metab. 2019;30(10):P745‐755. 10.1016/j.tem.2019.07.015 PMC6779524 31405774

