# Supplementary material for: A high fat, sugar, and salt Western diet induces motor‐muscular and sensory dysfunctions and neurodegeneration in mice during aging: Ameliorative action of metformin
Source: CNS Neurosci Ther. 2021 Sep 12;27(12):1458–71. doi: 10.1111/cns.13726 (PMC8611779; doi:10.1111/cns.13726)
Supplement: Supplementary file 1 — Supplementary Material [file CNS-27-1458-s002.docx]

**Supplemental Materials**

**Analysis of TNF-α in brain homogenates via enzyme-linked immunosorbent assay (ELISA)**

The brains were collected from C56BL/6 mice with or without the consumption of HFSS diet (from 20 to 22 months of age) and Met treatment immediately after decapitation under overdose anesthesia via ketamine/xylazine. One brain hemisphere of each mouse was used for histological study as described in the main text. The other brain hemisphere of each mouse was homogenized and lysed in the buffer provided with the TNFα ELISA kit and with addition of protease and phosphatase inhibitors (ThermoFisher). The lysate was centrifuged at 14,000 rpm for 25 min at 4°C. The total protein concentration in the supernatant was quantified using Bradford reagent (Bio-Rad). The TNFα level in the lysates were then quantified by an ELISA kit according to the manufacturer’s instructions (ThermoFisher).


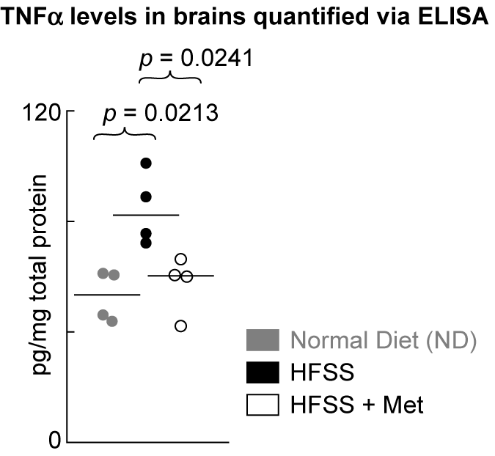
**Supplemental figure 1. Brain TNF-α levels during aging were increased by the chronic consumption of HFSS diet, and curbed by Met treatment**. TNF-α was measured via ELISA in brain homogenates of C56BL/6 (B6) mice with or without the consumption of HFSS diet from 20 to 22 months of age or Met treatment. Data are presented as dots and lines (medians). *p* ≤ 0.05 is significant (n = 4 mice).


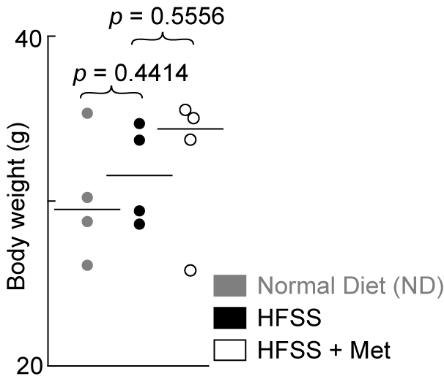
**Supplemental Figure 2.** Mouse body weights at the end of HFSS and metformin treatments

The mouse weights were measured. HFSS or metformin did not induce significant change of mouse body weight by the end of HFSS and metformin treatment (supplemental Figure 2). This may be related to the HFSS treatment duration that is close to the short side of the duration ranges for diet studies. Our results indicate that the behavior differences between normal diet (ND) and HFSS are not due to the body weight differences, and may be due to the differences including microglial phenotypes and the deposition of Aβ and pTau in the brains in motor and sensory cortexes (Figure 2 to 6). Our observation on 20 to 22 months old C57BL/6 mice is parallel to the report that 10 weeks of high fat diet did not cause significant change of body weights of 5xFAD mice, but accelerated the pathogenesis of Alzheimer’s disease like^37^.
