# Supplementary material for: A high fat, sugar, and salt Western diet induces motor‐muscular and sensory dysfunctions and neurodegeneration in mice during aging: Ameliorative action of metformin
Source: CNS Neurosci Ther. 2021 Sep 12;27(12):1458–71. doi: 10.1111/cns.13726 (PMC8611779; doi:10.1111/cns.13726)
Supplement: Supplementary file 2 — Supplementary Material [file CNS-27-1458-s001.docx]

**Supplemental figures 1 and 2 (see Supplemental Materials)**

Open Research: DATA AVAILABILITY STATEMENT. The data of this report are available from the corresponding author upon reasonable request.
